# Evidence for signatures of ancient microbial life in paleosols

**DOI:** 10.1038/s41598-020-73938-9

**Published:** 2020-10-08

**Authors:** Katharina Frindte, Eva Lehndorff, Stefan Vlaminck, Katharina Werner, Martin Kehl, Farhad Khormali, Claudia Knief

**Affiliations:** 1 Molecular Biology of the Rhizosphere, University of Bonn, Nussallee 13, 53115 Bonn, Germany; 2 Soil Ecology, Bayreuth University, Dr.-Hans-Frisch-Str. 1-3, 95448 Bayreuth, Germany; 3 Institute of Geography, University of Cologne, Albertus Magnus Platz, 50923 Köln, Germany; 4 Department of Soil Sciences, Gorgan University of Agricultural Sciences and Natural Resources, Gorgan, Iran

**Keywords:** Soil microbiology, Palaeoecology

## Abstract

Loess-paleosol sequences are terrestrial archives of past climate change. They may host traces of ancient microbial life, but little information is available on the recovery of microbial biomarkers from such deposits. We hypothesized that microbial communities in soil horizons up to an age of 127 kyr carry information related to past environments. We extracted DNA from a loess-paleosol sequence near Toshan, Northern Iran, with 26 m thick deposits showing different degrees of soil development, performed quantitative PCR and 16S rRNA gene amplicon sequencing. Periods of soil formation archived within the loess sediment led to higher diversity and bacterial abundance in the paleosol horizons. Community composition fluctuated over the loess-paleosol sequence and was mainly correlated with age and depth, (ADONIS *R*^2^ < 0.14, *P* ≤ 0.002), while responses to paleosol soil traits were weaker. Phyla like *Bacteriodetes*, *Proteobacteria* or *Acidobacteria* were more prevalent in paleosol horizons characterized by intense soil formation, while weakly developed paleosols or loess horizons hosted a higher percentage and diversity of *Actinobacteria*. Taken together, our findings indicate that the microbial community in loess-paleosol sequences carries signatures of earlier environmental conditions that are preserved until today.

## Introduction

Loess-paleosol sequences provide specific insight into climate change periodicity and intensity and therefore serve as archives of Earth history, giving detailed information e.g. about climate change effects on the biota and its environment at a regional scale^1–3^. Northern Iranian loess deposits are up to at least 70 m thick and have been formed by accumulation of aeolian dust. In the Late Quaternary these areas experienced periods of relatively humid and probably warm climatic conditions correlating with interglacials and interstadials, which have led to soil formation, whereas dry and probably cold climatic conditions of stadials promoted dust accumulation and burial of the paleosols^1,4^. Soil formation undergoes different successional stages leading to a steady enrichment of organic matter and more complex microbial colonization patterns over time^5–8^. Further aeolian dust deposition during successive colder or dryer climatic conditions led to the burial and conservation of these soils as relicts of past soil formation, being cut off from recycling by the overlying deposited loess sediment. The buried paleosol horizons are darker in color and enriched in organic matter^2,9^. Biogenic precursor materials, derived for example from higher plants, were identified in these paleosols^10^. It appears reasonable that also microbial cells and their DNA have been preserved and archive information about former microbial life.

So far, traces of earlier microbial life in loess or paleosols were primarily identified microscopically focusing on cyanobacteria, lichens and mosses^11,12^, or by cultivation-dependent methods to trace changes in the microbial community composition (MCC) formed during the last 4 kyr^13,14^. The use of cultivation-independent DNA-based molecular approaches bears potential to look further back into the past based on the detection and the analysis of the taxonomic information encoded by this marker molecule. This can extent our knowledge about past life and the environmental parameters that have influenced it. In recent years, microbiologists developed an interest in exploring environmental DNA from different natural archives to investigate microbial communities (MCs) with the aim to obtain information about present and ancient life^15–17^. Ancient microbial life has successfully been detected in aquatic sediments^18–20^ and permafrost soils^21–24^, as cold and dry conditions are thought to be best for the preservation of DNA in soil^15,21,22^. Dry terrestrial systems like buried paleosols in loess deposits may also serve as a valuable archive. While the upper, modern soil horizons can be expected to host a recent and metabolically active soil MC, microbial activity is probably substantially reduced in paleosols since their burial, so that these horizons may preserve microbial residues of the past. We hypothesize that DNA-based molecular approaches are suitable to trace the microbial history in loess deposits and its correlation with Quaternary climate periodicity in paleosols. Variation in the composition of microbial residues in different horizons of a loess paleosol sequence may serve as indicator for phases of soil formation under different climate conditions.

We traced the MCC by DNA-based approaches in loess and paleosols that were previously assigned to different age and climate periods^4,25,26^ up to a geological age of 127 kyr. We evaluated whether the identified microbes vary with depth and the sequence of soil horizons and whether they may represent residues of the MC prevalent during past soil formation phases. Microbial diversity, community composition and abundance were determined and correlated to data describing soil formation, such as accumulation of organic matter and weathering of minerals. This allowed us to identify potential drivers of community composition and abundance and to draw conclusions about the relevance of current and past factors that may have shaped the MC.

## Results

### Basic soil parameters and soil formation in the Toshan loess-paleosol sequence

The loess-paleosol sequence at Toshan is characterized by a modern soil on top (< 22.4 kyr ± 1.6) and ends with a strongly developed paleosol formed about 127 kyr ± 8 ago and buried at 25.6 m depth^26^ (Table S1). The different paleosol horizons developed under different climatic conditions such as precipitation and vegetation growth, and thus showed different characteristics, which can be used to reconstruct past climate^27,28^ (see supplementary information). Paleosol and loess horizons were previously identified by parameters indicating weathering and soil formation such as differences in grain size, redness index (RI), and magnetic properties^25–27^ (mass-dependent magnetic susceptibility, frequency dependent magnetic susceptibility, see Figure S1). These parameters were complemented by data for pH (H_2_O), total organic carbon (TOC) and nitrogen content (N) using the same soil samples in this study.

### MCC in the loess-paleosol sequence

**Figure 1:**
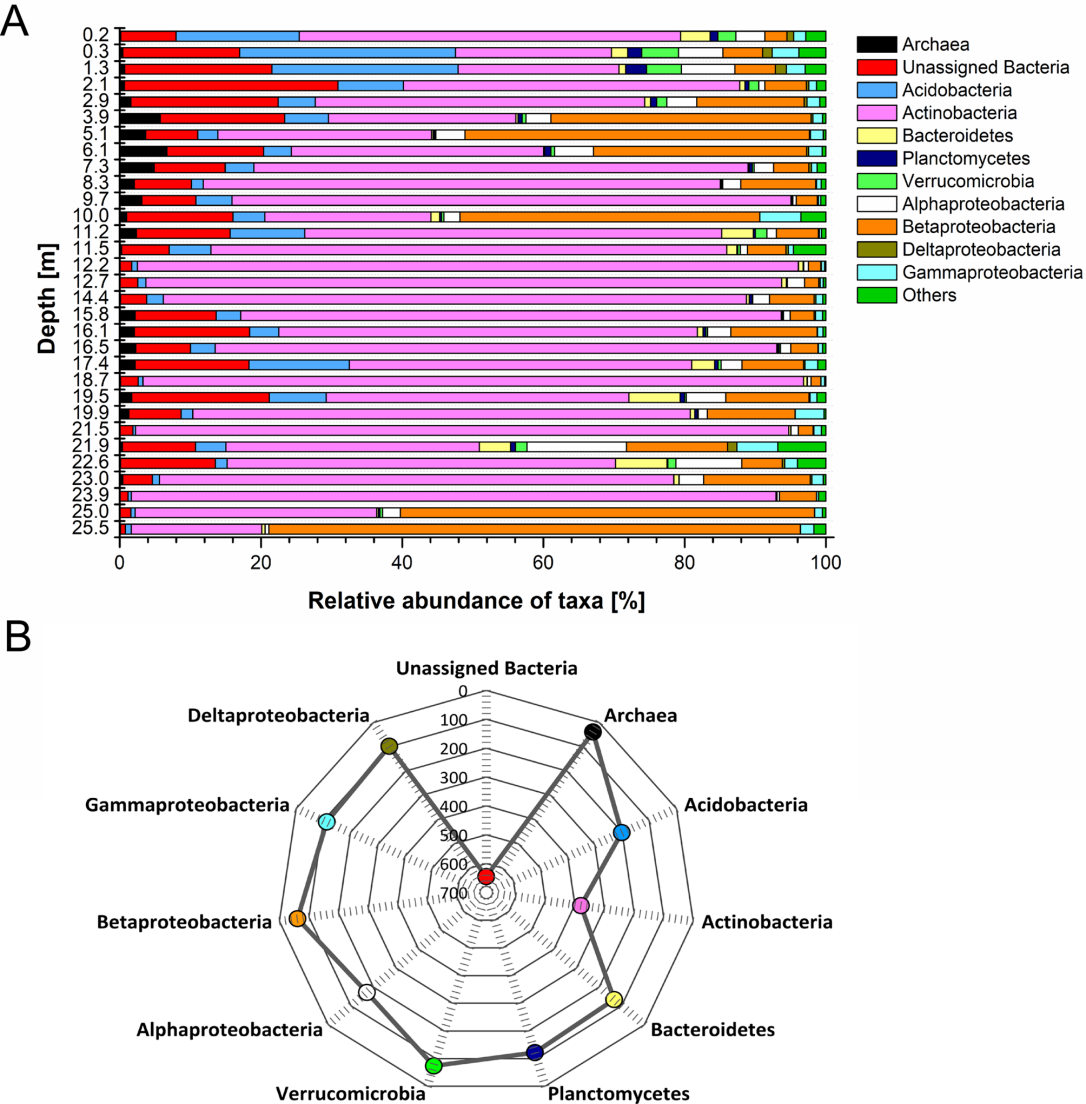
(**A**) Relative abundance of *Archaea,* different bacterial phyla and proteobacterial classes in the soil samples in relation to depth. (**B**) Number of different OTUs in the major taxonomic groups that were identified in the soil samples. Unclassified bacteria could not be assigned to a specific known bacterial species.

**Figure 2:**
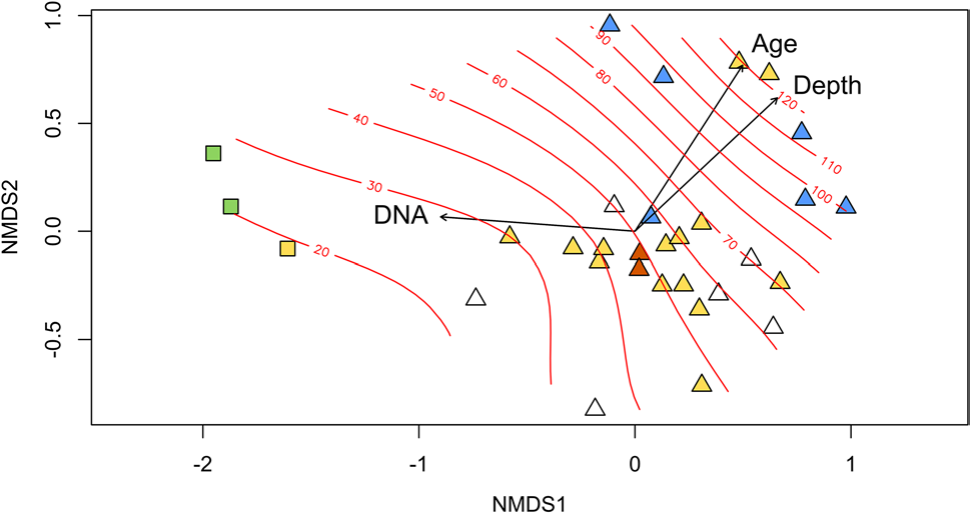
NMDS plot based on a Bray–Curtis distance matrix visualizing the differences in microbial community composition between samples with increasing age. The arrows were projected on the plot to visualize the impact of the three most significant parameters according to Envfit analysis, and contour lines were added to document the soil age in kyr. Squares show modern soil samples, while triangles indicate paleosols. Color code combines information about the degree of soil development (loess, weak, moderate and strong development) and climatic conditions: Light green color indicates the modern soil rich in organic matter; brown color indicates moderately developed horizons of synsedimentary paleosols formed under semiarid climatic conditions; yellow color indicates weakly developed synsedimentary paleosol horizons formed under arid to semiarid conditions; white color indicates loess horizons with nearly no pedogenic alteration under arid conditions; blue color indicates formation of postsedimentary paleosols during more humid climatic conditions.

16S rRNA gene amplicon sequencing revealed that the most abundant phyla in the microbial community were *Actinobacteria* (mean relative abundance and standard deviation: 58.1% ± 24) and *Proteobacteria* (20.1% ± 5.8), followed by *Acidobacteria* (5.9% ± 7.1, Fig. 1A). Within the proteobacterial clade, *Betaproteobacteria* (15.0% ± 18.2) and *Alphaproteobacteria* (3.2% ± 2.9) were predominant. *Planctomycetes* (0.5% ± 0.6), *Bacteriodetes* (1.5% ± 2.0), *Verrucomicrobia* (0.8% ± 1.2) and members of the domain *Archaea* (1.5% ± 1.7) were much less abundant. Among these, *Planctomycetes* and *Verrucomicrobia* were predominantly observed in the upper layers. The highest relative abundance of *Archaea* was found in layers between 3 and 11 m depth, while the relative abundance of *Bacteriodetes* varied over depth. We observed a high divergence in relative abundance of taxa between successive soil horizons (Fig. 1A). An evaluation of the microbial community composition at high taxonomic resolution based on operational taxonomic units (OTUs), which correspond roughly to species level resolution, revealed that the highest OTU diversity was seen within the phyla *Proteobacteria* (~ 500 OTUs), *Actinobacteria* (~ 400 OTUs) and *Acidobacteria* (~ 200 OTUs) (Fig. 1B). More than 600 OTUs were assigned as unclassified bacteria, i.e. their identity could not be further specified. Variation in the MCC between samples, visualized in a non-metric multidimensional scaling plot (NMDS; Fig. 2), confirmed the importance of soil depth and age, as samples separated well in relation to age and depth. Samples with a soil age up to 40 kyr (calendar years, determined by luminescence dating^26^) showed more variation along the first axis than older samples. The most deeply buried paleosols (> 90 kyr), which comprise mainly soils developed under humid climate conditions, were separated from most other samples along the second axis. Analyses of similarity (ANOSIM) was performed to relate MCC to the categorical factors soil horizon (*R* = 0.16, *P* = 0.08), degree of soil development (*R* = 0.11, *P* = 0.07) and climate conditions (*R* = 0.15, *P* = 0.06), but only little relatedness to these factors was found. The three modern soil horizons were clearly distinct from older soil horizons (ANOSIM, *R* = 0.72, *P* < 0.001), so that we assessed the impact of the categorical factors in addition by exclusion of these modern horizons, but this did not increase the impact of these factors.

To correlate the MCC data with the measured environmental parameters Envfit analysis was performed (Table S2). We observed a strong impact of soil age, soil depth, DNA content, TOC and N content. As TOC and N content were highest in modern soils, we excluded the modern soils and repeated the analyses, which resulted in non-significant correlations, demonstrating that the high correlation for TOC and N content was driven by differences in the modern soils (Table S2). Some soil traits such as median grain size and magnetic traits were weakly correlated to MCC data (*R*^2^ between 0.28 and 0.40, *P* < 0.05), while clay content, RI or pH were not correlated. To further assess the relevance of the main factors on MCC and potential autocorrelative effects between these, analyses of variance using distance matrices (Adonis) was performed. Age was the most significant factor (*R*^2^ = 0.13, *P* = 0.001; Table 1), followed by depth (*R*^2^ = 0.08, *P* = 0.002) and DNA content (*R*^2^ = 0.06, *P* = 0.005). Moreover, age was significantly autocorrelated with soil depth (*R*^2^ = 0.06, *P* = 0.007), but not with DNA content (*P* > 0.05).

**Table 1:** Results of ADONIS analysis (n = 31).

Parameter	*R*^2^	Significance
Depth	0.086	0.005
Age	0.133	0.001
DNA	0.061	0.005
Autocorrelation depth : age	0.063	0.006
Autocorrelation depth : DNA	0.072	0.002
Autocorrelation age : DNA	0.029	0.201
Autocorrelation depth : age : DNA	0.036	0.072

### Bacterial abundance, species richness and evenness

**Figure 3:**
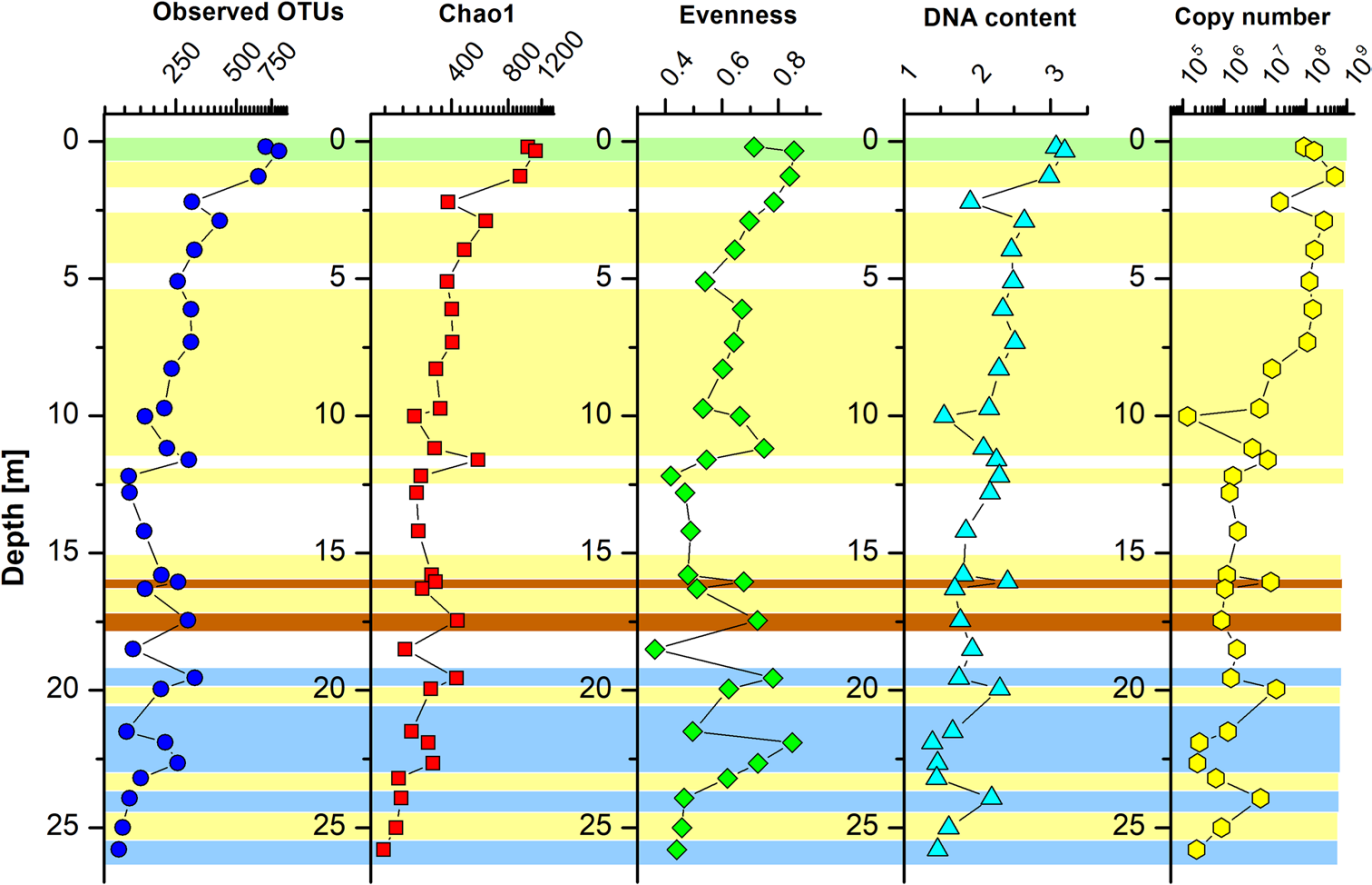
Observed changes of richness, evenness, DNA content [ng g^−1^ dry soil] and bacterial 16S rRNA gene copy number per g dry soil with increasing soil depth. Values for observed OTUs, Chao1 index and copy number are log_10_ scaled on the x-axis. The color code indicates horizons with different soil development and climate stages, likewise as used in Fig. 2.

Bacterial abundance, quantified by quantitative PCR (qPCR) of the 16S rRNA gene to estimate the absolute number of bacterial gene copies in the soil samples, decreased quite consistently with increasing soil depth (Pearson correlation coefficient *R* =  − 0.63, *P* ≤ 0.01, n = 31, Table S3) and correlated even better with soil age (*R* =  − 0.73, *P* ≤ 0.001, n = 31). Copy numbers were highest in the soil horizon at 1.3 m depth (~ 24 kyr) with up to 5 × 10^8^ gene copies per g dry soil (Fig. 3). Below 7.5 m, copy numbers never exceeded 1.9 × 10^7^ gene copies per g dry soil. Lowest copy numbers (< 10^6^ copies per g dry soil) were primarily detected in the loess horizons. Some of the deeper soil horizons (16 m, 20 m, 24 m) exhibited higher copy numbers compared to the respective overlaying horizons (Fig. 3). Besides, gene copy numbers correlated well with DNA content (Pearson correlation coefficient *R* = 0.66, *P* ≤ 0.001; n = 31).

The observed and total microbial richness, estimated by the Chao1 index, showed congruent patterns over soil depth. The Chao1 index reached nearly 1,000 OTUs in the modern soil (until 1.3 m depth) and decreased quite consistently with depth, down to 170–220 OTUs in the oldest horizons (> 100 kyr). Accordingly, Chao1 indices were well correlated with depth (*R* =  − 0.71, *P* ≤ 0.01, n = 31) and age (*R* =  − 0.61, *P* ≤ 0.001, n = 31; Table S3). However, local peaks of species richness were often seen in the strongly developed paleosols (17.5 m, 19.5 m, 21.9–22.6 m; Fig. 3). Chao1 indices were also strongly correlated with TOC and N content (even after exclusion of modern soil samples). In contrast, microbial evenness, which describes the species equilibrium in a sample, did not show clear trends with increasing depth or soil age and was not correlated with TOC or N content (*P* > 0.05). Both diversity estimates, Chao1 and evenness, were positively correlated with soil parameters that describe the state of soil formation (*R* between 0.46 and 0.62, *P* ≤ 0.01, n = 31; Table S3), i.e. magnetic susceptibility, frequency-dependent magnetic susceptibility (i.e. abundance of magnetic minerals that formed during soil formation) and RI (i.e. formation of reddish mineral phases during soil formation).

### Changes in the relative abundance of OTUs over depth

**Figure 4:**
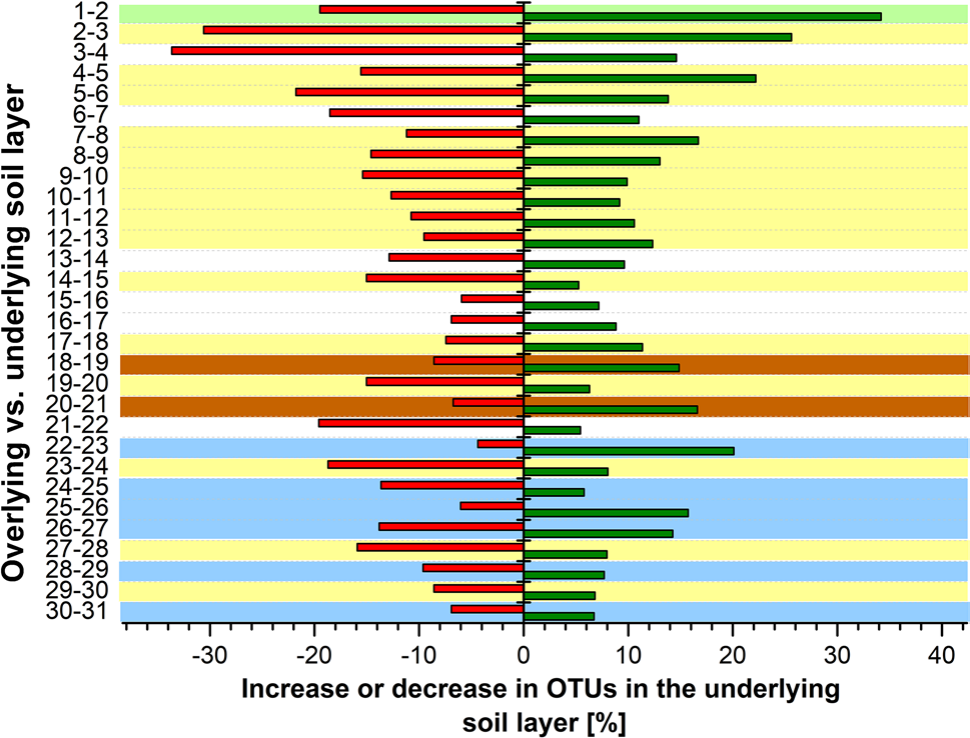
Percentage of OTUs that decreased (red) or increased (green) > 15% in relative abundance in a soil horizon compared to the respective overlying horizon. The percentage of increased or decreased OTUs was calculated in relation to the total number of all OTUs (1144). The y-axis denotes each pair of successive soil horizons which are compared using the soil horizon numbers according to Table S1 The color code given for each comparison indicates the soil development and climate stages of the respective underlying soil horizon according to Fig. 2.

A good preservation of ancient community structures would be evident from clear shifts between successive horizons. Therefore, we determined the number of OTUs that show increasing or decreasing relative abundances of more than 15% between successive soil horizons (Fig. 4). In case of a strong responsiveness of the MCC to the vertical structuring of soil horizons, we expected to observe fluctuations primarily in transitions from loess or weakly developed horizons to underlying strongly developed paleosols due to limited translocation from loess horizons to underlying soils under arid conditions. In contrast, weaker changes were expected in transitions from paleosols to underlying loess layers due to possible vertical translocation by water during the more humid periods. The most intensive changes between successive horizons occurred in the modern soil horizons (horizon numbers 0–4), pointing towards a strong depth-dependent stratification of the active microbiota. Between horizons 5 and 17, the percentage of increasing and decreasing OTUs declined quite consistently until a minimum between horizons 15 and 16 (Fig. 4), where a transition from a paleosol to loess occurred. In the deeper horizons with more intense soil formation, the percentage of OTUs fluctuating between the successive soil horizons increased again, especially between horizons 18–19 and 20–21 and 22–23, where well developed paleosols are overlain by weakly developed paleosols or loess, and additionally between horizons 25 and 26, thus largely confirming our expectations.

**Figure 5:**
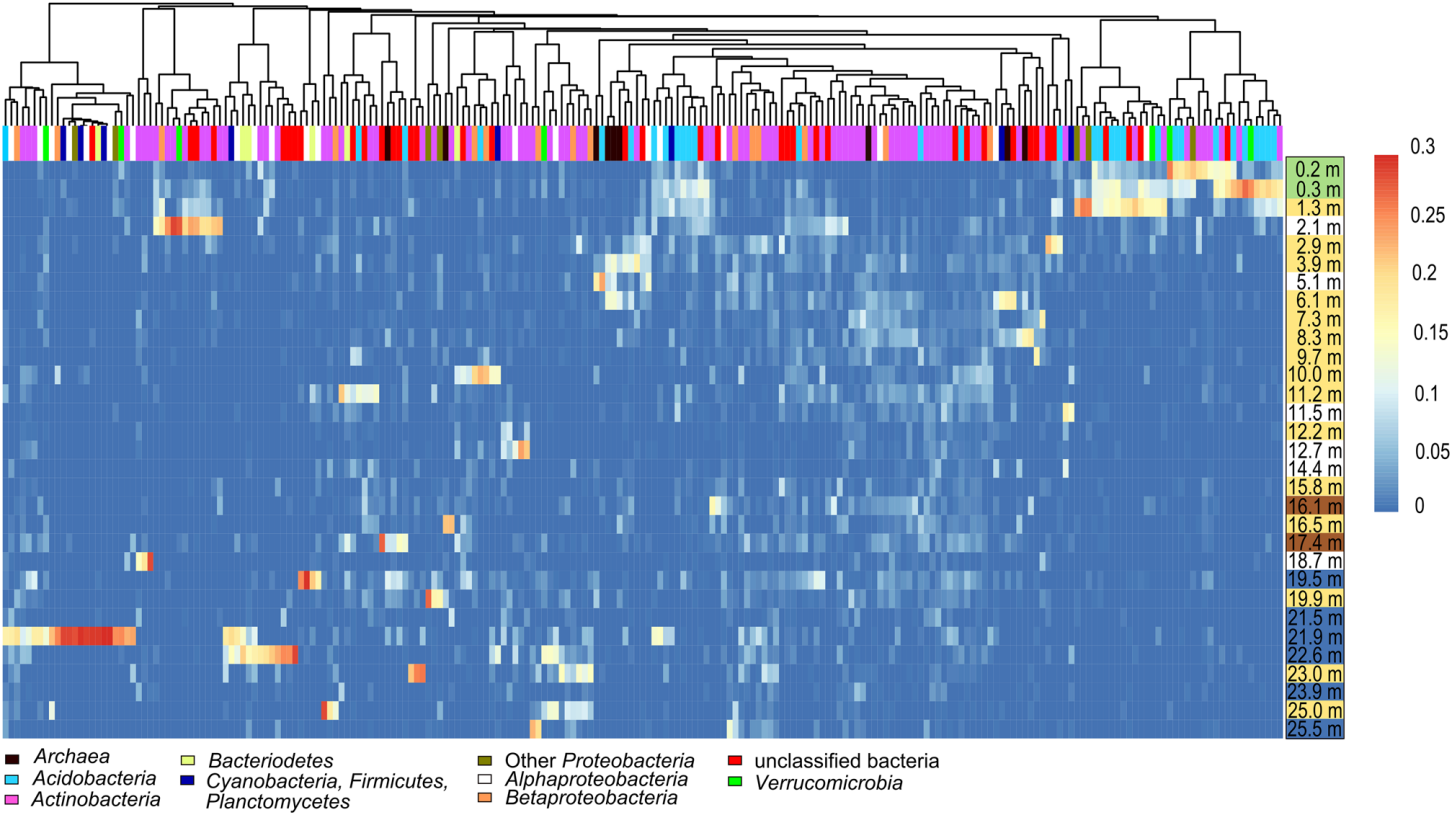
Log_10_ transformed heatmap showing the preferential occurrence of the most abundant OTUs (> 0.5% relative abundance) across soil depth. The relative abundance of each OTU was normalized to 1 to adjust the patterns of all OTUs. The dendrogram groups OTUs with similar distribution patterns across depth. Complete clustering of OTUs was done based on an Euclidean distance matrix. The color code of the depth profile indicates different soil development and climate stages of soil horizons according to Fig. 2.

An evaluation of the distribution of OTUs across depth revealed that only few OTUs, which were affiliated to different phyla, were present at all depths (Fig. 5). Different members of *Acidobacteria* and *Actinobacteria* occurred almost exclusively in the modern soil, while OTUs of some other non-actinobacterial phyla or domains dominated in deeper horizons (e.g. archaeal OTUs between 3 and 6 m). A particular enrichment of specific OTUs occurred in paleosol horizons that are buried below 16 m depth (especially at 21.9 m) and developed under more humid conditions with OTUs belonging to the phyla *Proteobacteria*, *Acidobacteria*, *Verrucobacteria*, *Planctomycetes*, *Firmicutes* and *Bacteriodetes*. Many OTUs of the phylum *Actinobacteria* were rather evenly distributed over the whole profile, but especially between 3 and 20 m depth. For further details about the distribution of specific OTUs see supplement (Figure S2 and additional text).

## Discussion

### Variation in microbial diversity, abundance and composition due to soil depth and former soil development

We successfully extracted environmental DNA from different horizons of an arid paleosequence consisting of modern soil, loess and paleosol horizons reaching a maximum estimated soil age of 127 kyr. In general, DNA yield decreased with increasing depth, but we detected variations of DNA yield in different soil horizons, which correlated well with bacterial copy numbers. This indicates that the extracted DNA was mainly derived from bacteria. Likewise, we observed a good correlation between depth or soil age and alpha diversity indices, as all diversity estimates showed a decreasing trend with increasing depth/age (Fig. 3). This overall decline in alpha diversity and abundance is well known from studies of other soil profiles, which analyzed profiles up to 20 m deep^29–32^. It is primarily reflecting the decreasing microbial population size with increasing depth. In addition, it may be affected by the quality of the DNA of buried microorganisms (as discussed below).

The decreasing trend of bacterial community traits with depth was interrupted by deviations in the paleosol horizons, which hosted a higher bacterial abundance and diversity. This becomes especially obvious for the horizon at 21.9 m depth showing an evenness index as high as in the modern soils. In comparison, horizons with weak indication of soil formation and loess horizons usually showed lowest richness and evenness values (Fig. 3). Furthermore, microbial richness and evenness were significantly related to physico-chemical parameters describing soil formation processes (TOC, RI, magnetic properties; Table S3), thus indicating a correlation with the soil development. Taken together, these findings reveal that advanced soil formation is accompanied by a more abundant and diverse microbiota. We expected to see a clear dependency also between organic carbon contents and microbial diversity and abundance, as reported for soil layers buried for decades^31^, because microbial life in soil is primarily supported by organic carbon, but such a relationship was not seen for abundance, only for the Chao1 diversity estimate.

Similar to alpha diversity, MCC changed significantly with increasing depth and soil age (Fig. 2), but with weaker or no correlation to the specified soil horizons, degree of soil development, paleoclimatic conditions and the physico-chemical soil parameters (Table S2). Primarily, a clear separation of the modern soil horizons and of nearly all old horizons (> 90 kyr) was evident (Fig. 2). Recent studies of the loess-paleosol sequence in Toshan reported that the climate in this region was more arid during soil formation phases until ~ 70 kyr ago, while older paleosols till ~ 127 kyr were formed in a more humid climate^4,26,33^. Therefore, the differences in MCC in the oldest samples are probably related to the well-developed soils that have formed under a more humid climate. Besides this large-scale pattern, a very specific correlation to past climatic conditions or specific soil horizons remained weak and just below the limit of being significant, even when we removed the modern soil layers in our analyses (data not shown). Possibly, changes in MCC occurring after burial have blurred these signatures. It thus appears that the preservation of paleoenvironmental signatures in the MCC of this loess-paleosol sequence is weaker compared to findings from some permafrost, aquatic or subseafloor sediments^34–36^.

In summary, changes in bacterial abundance, diversity and community composition were found and observed to be primarily correlated to soil depth and age. However, deviations from the overall decline in abundance, diversity and in MCC were evident and moderately correlated to specific soil traits that describe soil development, indicating that soil formation promoted microbial diversity in former times and that these patterns are at least partially archived in the loess-paleosol sequence until today.

### Differentiation of modern and ancient soil microbial communities in buried soil horizons

To identify and reconstruct former microbial life, it is important to know whether a preservation of ancient microorganisms occurred upon burial, and if we can detect these via DNA analysis. In principal, total DNA extraction from soil allows the detection of both, ancient and present-day microorganisms. The ancient MC is detected based on ancient (old DNA, extracellular), relic (ancient intracellular or extracellular DNA from dead cells) and DNA of viable but probably dormant cells^16,37^. Several studies suggest that DNA in soil can survive 0.5–1 million years, but this depends on the storage conditions, e.g. the contents of humic acids, temperature, water and clay content, as well as ionic strength and pH^16,17,24,37,38^. In our soils low oxygen contents, constant temperatures, high clay contents and low moisture conditions might be the main factors that support the preservation of the MC. High pH values as measured in the buried soil horizons are considered negative for preservation, but may play a minor role here, as the water content is very low (< 15%). Thus, we consider that the conditions for preservation are in general advantageous. Nevertheless, especially the extracellular DNA will undergo degradation over time, so that ancient DNA is usually fragmented and therefore increasingly difficult to amplify by PCR upon aging^16^. An accumulation of fragmented DNA should be detectable in the DNA extracts, but we did not observe large amounts of partially degraded DNA in agarose gels (Figure S3), especially not with increasing depth and thus age, suggesting that degraded DNA was not increasingly contributing to the amount of recovered DNA with increasing soil depth. Thus, we assume that the DNA extracted from the paleosols is mainly derived from dormant or viable cells, which may represent at least in part left-overs from buried ancient MCs, or from extracellular DNA, which has not yet been degraded. Favorable environmental conditions in the paleosols may have prevented extracellular DNA from degradation so far, but to verify this, further studies are necessary.

Further evidence for the detection of traces of former microbial life in the buried soil horizons is derived from the depth-related changes in the MCC. Despite the fact that a certain arrangement of the samples according to depth was observed in the NMDS plot (Fig. 2), the more detailed analysis at OTU level revealed quite drastic changes in the MCC within the loess-paleosol sequence between different depth horizons (Fig. 4), indicating that quite distinct bacterial communities developed in these horizons. Moreover, we observed strong fluctuations in bacterial diversity, evenness and abundance between some of the successive horizons in the loess-paleosol sequence and enhanced diversity and abundance at specific depths. These strong changes indicate limited exchange of microorganisms between successive horizons in the profile and discontinuities in the development of MCs in successive horizons. Especially the clear differences in MCs between weakly developed horizons and underlying paleosols can reflect (a) differences in the MCC at the time the soil was buried, or (b) a present-day community, which developed over time upon burial, because we cannot exclude that life and metabolic activity continued at a low level, leading to a different MC. We only expect very limited metabolic activity or even growth in the deeper buried horizons due to the lack of water. Microbial activity in soil demands a minimum of available water, not only to maintain the hydration of the cells, but also to sustain a flow of nutrients towards the cells^39–41^. Further, it should be noted that the buried microorganisms in a specific horizon represent an isolated source community for the further development to a present-day MCC.

At some depths, we detected a maximum of microbial richness and diversity in the weakly developed soil horizons directly below a paleosol horizon. This appears contradictory, as highest diversity was expected to be seen in the most strongly developed soil horizons. However, cells may have been translocated from the above paleosol horizon into the underlying horizon by vertical water flow^42^. We assume that this leaching has occurred primarily during the more humid phases at the time of active soil formation, because water flow due to present-time rainfall does not reach deeper soil horizons^9^. Besides, the absorption of microbial cells to soil particles is intensified by low water availability, which reduces cell metabolism and supports immobility^42^. Assuming that the microbial patterns between paleosols and underlying loess horizons formed indeed predominantly in the past when the paleosols were surface exposed and water was reaching the underlying loess, and considering that we detected these patterns suggests that a preservation of patterns has occurred and therewith some conservation of the MC over time. Such similarities between MCC in successive horizons representing different levels of soil development were especially seen in the weakly developed soil horizons in the deeper horizons, which clustered together in the NMDS plot with paleosols from successive horizons (> 90 kyr; Fig. 2). In summary, the various findings and their interpretation suggest that the MCC shows patterns over depth that may have formed in the past and have been preserved in loess and paleosols until today to some extent, especially in the oldest part of the profile studied here.

### Do taxa which are presumed members of old MCs provide information about past processes?

With the aim to identify characteristic taxa for specific soil horizons, we had a closer look at the OTUs that occurred distinctly in modern soils, loess and paleosols. In a recent study, microbial communities of a vertical loess profile until 20 m from the semi-arid loess plateau in China covered with *Pinus* or *Robinia* forests were analyzed, showing that *Acidobacteria*, *Actinobacteria* and *Proteobacteria* were the predominant phyla over all depths^32^. In our study, acidobacterial OTUs (especially belonging to GP6, GP7, GP16; Fig. 5 and Figure S2) were primarily present in the modern soil horizons, suggesting that a major part of these organisms might not survive burial. *Acidobacteria* are usually abundant in soils worldwide, though not consistently in desert soils, leading to the suggestion that the alkaline pH or the very low water or carbon availability might be important controls for the members of this phylum^43–46^. In contrast, some other taxa were present in modern soil horizons and paleosols. This applies to verrucomicrobial OTUs of which most were found in the upper soil horizons, while other OTUs were found deeply buried in a paleosol horizon with intense soil formation (21.9 m), but not in horizons in between (Fig. 5). A strong decrease in the relative abundance of *Verrucomicrobia* in buried soil horizons up to an age of 3.7 kyr was also reported in a recent study^47^. We also observed specific enrichments of non-actinobacterial OTUs in paleosols, e.g. *Methylotenera, Methylobacterium, Oxalobacteriaceae, Erythrobacteriaceae, Ohtaekwangia* and *Phytomonospora* (Figure S2). All these organisms are often found in surface soils and/or in association with plants^48–52^. This can be taken as indication that parts of the original MC may have survived in these soils or at least their DNA remained detectable.

Loess horizons revealed a tendency to an impoverishment of the MC compared to paleosols with usually lowest Chao1 and evenness indices (especially between 5 and 14 m), fitting well to our understanding of loess as aeolian sediment mostly poor in life, which has been deposited during glacial periods. We found high actinobacterial abundances in both, loess and paleosols, although *Actinobacteria* appeared to be especially predominant in weakly developed soil horizons. This is consistent with recent studies which report that *Actinobacteria* were abundant in semi-arid loess soils^32,53^ and more abundant under alkaline soil conditions^53^, as in our study. *Actinobacteria* with high relative abundances were *e.g. Nocardioides*, *Arthrobacter*, *Amylocolatopsis, Pseudonocardia, Kribella* and *Streptomycetes*, which are known to occur frequently in arid desert soils^54,55^. Other actinobacterial OTUs assigned to *Glycomyces, Stackebrandtia, Saccharipolyspora* and *Gaiella* have not yet been described as typical soil microorganisms of arid environments, but seemed to be highly abundant in Iranian soil horizons regardless of the soil developmental stages. Overall, we observed a very high diversity of actinobacterial OTUs, likewise as described for desert soils^45,56,57^, which raises the question of the origin of these organisms. It is conceivable that *Actinobacteria* were introduced via dust transport in parallel with the deposition of loess. Polymenakou et al. have shown that intense African dust storms can transport *Actinobacteria* in low particle size dust fractions (< 3.3 µm)^58^. In the study area at Toshan, desert microorganisms may be introduced by near-surface winds, which frequently introduce silt-sized materials from the neighbored arid Caspian Lowland (approx. 1200 km) and possibly also the Karakum Desert (approx. 550 km). Such dust transport pathways were probably active during the past, as suggested for the area of the so-called Iranian Loess Plateau, located about 100 km to the east of the Toshan section^59^. However, we did not find the same actinobacterial OTUs over the whole soil depth profile, which suggests that colonization of surface-exposed horizons occurred from different sources, as loess originates from different regions, or that conditions varied over time also in the source regions, resulting in the introduction of different actinobacterial genera in different horizons.

Remarkably, we also found an OTU assigned to *Limnobacter thiooxidans* at high relative abundance in horizons with different soil developmental stages between 2.5 and 7.5 m and again below 24 m. This organism is a typical inhabitant of freshwater or brackish water sediments and was also found in high abundances in Caspian sea sediment samples^60–62^. The detection of this taxon might be another indicator for changing dust sources in the past, whereby dust events potentially translocated bacteria from the coastal lowland of the Caspian Sea to the Toshan region.

Regarding the distribution of particular OTUs across very specific depth layers of the soil profile we are cautious with more detailed interpretations, as it needs to be kept in mind that this first study was mainly explorative and therefore performed without sample replicates for each depth. In order to draw more solid conclusions about specific distribution patterns of selected taxa, and likewise about particular differences between adjacent depth layers, a more comprehensive study with replicated depth profiles would be valuable, even if it will be substantial effort to generate truly independent depth profiles. Notwithstanding this, the distribution patterns of the specific OTUs discussed here appear conclusive in the context of the biological knowledge of these taxa. Likewise, the repeated occurrence of specific soil horizons or climatic conditions over depth can be considered as a kind of replicate and gives more confidence. Thus, our data provide in sum different pieces of evidence that members of old and buried MC remain at least in part detectable.

## Conclusion

In this study we collected evidence for the detection of ancient microbial life in old loess-paleosol sequences using DNA as molecular marker. As a prerequisite, we detected predominantly DNA of microbial origin in all soil horizons up to 127 kyr. We observed trends of decreasing bacterial diversity and abundance with increasing depth, but this trend was interrupted by local peaks of increased abundance and diversity, especially in deeper soil horizons. In addition, we observed quite strong shifts in MCC with depth. These patterns are at least in part driven by the soil developmental status of the different horizons, as we observed correlations between MCC, microbial abundance and diversity with soil properties. Further evidence for the detection of former microbial life stems from the detection of specific bacterial taxa, i.e. paleosols with taxa that are typically known from above-ground habitats (plants), or surface soils. In contrast, loess horizons hosted a high diversity and abundance of *Actinobacteria*, which are well known to dominate in desert soils. Their origin can be well explained by aeolian transport and subsequent deposition, along with dust from nearby deserts. Taken together, we are confident that we found remnants of the ancient microbiota in the loess-paleosol sequence at Toshan, although the differentiation between ancient and present-day members in the detected MC remains a challenging task to be addressed in more detail in future studies. A differential analysis of extracellular versus intracellular DNA and analysis of short fragments of ancient DNA could be useful to address this in more detail. We propose that loess-paleosol sequences in arid environments can be used as terrestrial archives to obtain information about former microbial life and relate it to soil formation, past climate conditions or the source regions of loess. Moreover, understanding soil microbiology along with past climate change can be very valuable to predict effects of future changes. Long-term studies forecast overall reduced precipitation in this region^63,64^, suggesting that similar changes in the soil microbiota may develop as already archived in the loess-paleosol sequences under study.

## Material and methods

### Sampling site

Samples were taken from a loess-paleosol sequence in the Toshan area (N 36°49′01″/E 54°25′25″) in Golestan Province, Northern Iran. Here soils formed under different climates of the past in loess, a fine-grained aeolian deposit. For sampling of fresh, uncontaminated material five staircases were digged reaching about 0.5–3.5 m deep into the vertical exposure walls of the loess deposit^25^ (see supplementary material and Figure S4). TOC and N contents were measured for this study using a MicroCube elemental analyzer (Elementar, Hanau, Germany) according to DIN ISO 10,694. Before TOC analysis carbonates were removed using HCl (15%). The pH was measured in 10 g of dry soil with 25 ml of H_2_O. Parameters for soil formation and indicators for different climatic periods, age, volumetric median of grain size distribution, clay content, RI, mass specific magnetic susceptibility and frequency dependent magnetic susceptibility were measured in previous studies^4,25,26^ (more detail in supplemental information, and Figure S1).

### DNA extraction, quantification and purification

DNA was extracted from 3.75 g homogenized soil per sample using a phenol–chloroform based protocol and finally dissolved in 300 µL of PCR grade water^65^. Further details about DNA extraction are given in the supplementary material. The total DNA content in the extracts was quantified using the QuantiFluor dsDNA system (Promega, Mannheim, Germany) and DNA quality evaluated on 1.5% agarose gels. Prior to PCR, DNA underwent further purification steps (see supplementary information).

### Amplicon sequencing and qPCR

PCRs for Illumina amplicon sequencing were performed with primer pair 515F/806R^66^ in a two-step procedure as described in detail in the supplementary material. The obtained PCR-products were quantified, pooled at equimolar concentrations and purified with HighPrep magnetic beads (Biozym, Hessisch Oldendorf, Germany). Library preparation and sequencing on an Illumina HiSeq system in 2 × 250 bp rapid run mode was done by the Max Planck-Genome-centre Cologne (further details in the supplementary information). Raw sequence reads have been deposited in the European Nucleotide Archive (PRJEB35011).

The abundance of bacteria was determined by qPCR targeting the 16S rRNA gene with primers Bac349f./Bac806r^67^. Further details are provided in the supplementary material.

### Bioinformatics data processing

Sequence data were assembled with the USEARCH paired-read assembler^68^ to create sequences with a consensus of at least 90% and a quality score of Q = 2. Sequences were trimmed, demultiplexed, quality checked and binned into OTUs as described before^69^. Corrections for PCR negative controls and DNA extraction blanks were performed as described in the supplement.

### Statistical analyses of MC data

Data analysis was performed in R^70^ using the package vegan^71^. NMDS plots were calculated using a Bray–Curtis distance matrix based on a rarefied OTU table (4121 reads). The influence of the categorical variables soil horizon, degree and type of soil development and prevailing climate conditions (Table S1) was tested by ANOSIM. Envfit analyses were applied to evaluate the impact of metric environmental variables (age, depth, pH, RI, mass specific magnetic susceptibility, frequency dependent magnetic susceptibility, grain size, clay content, DNA content, TOC and N) on MCC. The most influencing factors (*R* > 0.70) were chosen for Adonis.

### Statistical analysis of qPCR data, estimated microbial richness and evenness

Diversity indices were calculated in R-studio with the package vegan^70,71^. Observed OTUs, Chao1, and Pilou’s evenness were calculated from the rarefied OTU table. Kruskal–Wallis tests were used to identify significant differences in Chao1, evenness and 16S rRNA gene copy numbers (after log_2_ transformation) between groups of samples, defined according to the categorical variables. To explore the impact of the metric environmental variables on copy numbers, Chao1 index and evenness, we performed Pearson correlation analysis. *P*-values in analyses with multiple comparisons were corrected by applying the Bonferroni-Holm procedure.

## Supplementary information

Supplementary file1.

Supplementary file2.

## References

[1] Kehl, M. *Quaternary Loesses, Loess-Like Sediments, Soils and Climate Change in Iran* (Gebrüder Borntraeger Verlagsbuchhandlung, 2010).

[2] Kehl M, Sarvati R, Ahmadi H, Frechen M, Skowronek A (2005). Loess paleosol-sequences along a climatic gradient in Northern Iran. Eiszeitalt. Ggw..

[3] Bradley RS (1999). Paleoclimatology: Reconstructing Climates of the Quaternary.

[4] Vlaminck S (2018). Late Pleistocene dust dynamics and pedogenesis in Southern Eurasia—Detailed insights from the loess profile Toshan (NE Iran). Quat. Sci. Rev..

[5] Schulz S (2013). The role of microorganisms at different stages of ecosystem development for soil formation. Biogeosciences.

[6] Tscherko D, Rustemeier J, Richter A, Wanek W, Kandeler E (2003). Functional diversity of the soil microflora in primary succession across two glacier forelands in the Central Alps. Eur. J. Soil Sci..

[7] Nemergut DR (2007). Microbial community succession in an unvegetated, recently deglaciated soil. Microb. Ecol..

[8] Turner S (2017). Microbial community dynamics in soil depth profiles over 120,000 years of ecosystem development. Front. Biol..

[9] Chaopricha NT, Marín-Spiotta E (2014). Soil burial contributes to deep soil organic carbon storage. Soil Biol. Biochem..

[10] Shahriari A (2017). Biomarkers in modern and buried soils of semi-desert and forest ecosystems of northern Iran. Quat. Int..

[11] Svirčev Z (2013). Importance of biological loess crusts for loess formation in semi-arid environments. Quat. Int..

[12] Dulić T (2017). Cyanobacterial diversity and toxicity of biocrusts from the Caspian Lowland loess deposits, North Iran. Quat. Int..

[13] Demkina TS, Khomutova TE, Kashirskaya NN, Stretovich IV, Demkin VA (2009). Characteristics of microbial communities in steppe paleosols buried under kurgans of the Sarmatian time (I-IV centuries AD). Eurasian Soil Sci..

[14] Khomutova TE (2007). An assessment of changes in properties of steppe kurgan paleosoils in relation to prevailing climates over recent millennia. Quat. Res..

[15] Thomsen PF, Willerslev E (2015). Environmental DNA: an emerging tool in conservation for monitoring past and present biodiversity. Biol. Conserv..

[16] Pedersen MW (2015). Ancient and modern environmental DNA. Philos. Trans. R. Soc. B.

[17] Bálint M (2018). Environmental DNA time series in ecology. Trends Ecol. Evol..

[18] Coolen MJL (2004). Combined DNA and lipid analyses of sediments reveal changes in Holocene haptophyte and diatom populations in an Antarctic lake. Earth Planet. Sci. Lett..

[19] Monchamp M-E (2018). Homogenization of lake cyanobacterial communities over a century of climate change and eutrophication. Nat. Ecol. Evol..

[20] Belle S (2014). Temporal changes in the contribution of methane-oxidizing bacteria to the biomass of chironomid larvae determined using stable carbon isotopes and ancient DNA. J. Paleolimnol..

[21] Bellemain E (2013). Fungal palaeodiversity revealed using high-throughput metabarcoding of ancient DNA from arctic permafrost. Environ. Microbiol..

[22] Zhang DC, Brouchkov A, Griva G, Schinner F, Margesin R (2013). Isolation and characterization of bacteria from ancient Siberian permafrost sediment. Biology (Basel).

[23] Gilichinsky D, Margesin R, Schinner F, Marx J-C, Gerday C (2008). Bacteria in permafrost. Psychrophiles: From Biodiversity to Biotechnology.

[24] Willerslev E (2004). Long-term persistence of bacterial DNA. Curr. Biol..

[25] Vlaminck S (2016). Loess-soil sequence at Toshan (Northern Iran): insights into late Pleistocene climate change. Quat. Int..

[26] Lauer T (2017). Luminescence-chronology of the loess palaeosol sequence Toshan, Northern Iran: a highly resolved climate archive for the last glacial-interglacial cycle. Quat. Int..

[27] Khormali F, Kehl M (2011). Micromorphology and development of loess-derived surface and buried soils along a precipitation gradient in Northern Iran. Quat. Int..

[28] Khormali F, Ghergherechi S, Kehl M, Ayoubi S (2012). Soil formation in loess-derived soils along a subhumid to humid climate gradient, Northeastern Iran. Geoderma.

[29] Fierer N, Schimel JP, Holden PA (2003). Variations in microbial community composition through two soil depth profiles. Soil Biol. Biochem..

[30] Eilers KG, Debenport S, Anderson S, Fierer N (2012). Digging deeper to find unique microbial communities: the strong effect of depth on the structure of bacterial and archaeal communities in soil. Soil Biol. Biochem..

[31] Helgason BL, Konschuh HJ, Bedard-Haughn A, VandenBygaart AJ (2014). Microbial distribution in an eroded landscape: Buried A horizons support abundant and unique communities. Agric. Ecosyst. Environ..

[32] Liu G (2020). Vertical changes in bacterial community composition down to a depth of 20 m on the degraded Loess Plateau in China. Land Degrad. Dev..

[33] Lauer T (2017). The Agh Band loess-palaeosol sequence—A terrestrial archive for climatic shifts during the last and penultimate glacial–interglacial cycles in a semiarid region in northern Iran. Quat. Int..

[34] Mitzscherling J (2019). Microbial community composition and abundance after millennia of submarine permafrost warming. Biogeosci. Discuss..

[35] Vuillemin A, Ariztegui D, Leavitt PR, Bunting L (2016). Recording of climate and diagenesis through sedimentary DNA and fossil pigments at Laguna Potrok Aike, Argentina. Biogeosciences.

[36] Ciobanu M-C (2012). Sedimentological imprint on subseafloor microbial communities in Western Mediterranean Sea Quaternary sediments. Biogeosciences.

[37] Carini P (2016). Relic DNA is abundant in soil and obscures estimates of soil microbial diversity. Nat. Microbiol..

[38] Drancourt M, Raoult D (2005). Paleomicrobiology: Current issues and perspectives. Nat. Rev. Microbiol..

[39] Stevenson A (2015). Multiplication of microbes below 0.690 water activity: implications for terrestrial and extraterrestrial life.. Environ. Microbiol..

[40] Schimel JP (2018). Life in dry soils: Effects of drought on soil microbial ommunities and processes. Annu. Rev. Ecol. Evol. Syst..

[41] Lebre PH, De Maayer P, Cowan DA (2017). Xerotolerant bacteria: Surviving through a dry spell. Nat. Rev. Microbiol..

[42] Joergensen RG, Wichern F (2018). Alive and kicking: Why dormant soil microorganisms matter. Soil Biol. Biochem..

[43] Aslam SN (2016). Soil compartment is a major determinant of the impact of simulated rainfall on desert microbiota. Environ. Microbiol..

[44] Armstrong A (2016). Temporal dynamics of hot desert microbial communities reveal structural and functional responses to water input. Sci. Rep..

[45] Knief C (2020). Tracing elevational changes in microbial life and organic carbon sources in soils of the Atacama Desert. Glob. Planet. Change.

[46] Kielak AM, Barreto CC, Kowalchuk GA, van Veen JA, Kuramae EE (2016). The ecology of *Acidobacteria*: moving beyond genes and genomes. Front. Microbiol..

[47] Chernov TI (2018). Comparative analysis of the structure of buried and surface soils by analysis of microbial DNA. Microbiology.

[48] Knief C, Ramette A, Frances L, Alonso-blanco C, Vorholt JA (2010). Site and plant species are important determinants of the *Methylobacterium* community composition in the plant phyllosphere. ISME J..

[49] Fierer N, Colman BP, Schimel JP, Jackson RB (2006). Predicting the temperature dependence of microbial respiration in soil: A continental-scale analysis. Glob. Biogeochem. Cycles.

[50] Baldani JI, Rosenberg E, DeLong EF, Steckebrandt E, Thompson F (2014). The family *Oxalobacteraceae*. The Prokaryotes: Alphaproteobacteria and Betaproteobacteria.

[51] Li J (2011). *Phytomonospora endophytica* gen. nov., sp. nov., isolated from the roots of *Artemisia annua* L.. Int. J. Syst. Evol. Microbiol..

[52] Eyice Ö (2015). SIP metagenomics identifies uncultivated *Methylophilaceae* as dimethylsulphide degrading bacteria in soil and lake sediment. ISME J..

[53] Liu D, Yang Y, An S, Wang H, Wang Y (2018). The biogeographical distribution of soil bacterial communities in the Loess Plateau as revealed by high-throughput sequencing. Front. Microbiol..

[54] Trujillo ME (2017). *Pseudonocardia nigra* sp. nov., isolated from Atacama desert rock. Int. J. Syst. Evol. Microbiol..

[55] Mohammadipanah F, Wink J (2016). *Actinobacteria* from arid and desert habitats: diversity and biological activity. Front. Microbiol..

[56] Goodfellow M, Nouioui I, Sanderson R, Xie F, Bull AT (2018). Rare taxa and dark microbial matter: novel bioactive actinobacteria abound in Atacama Desert soils. Antonie van Leeuwenhoek.

[57] Bull AT (2018). High altitude, hyper-arid soils of the Central-Andes harbor mega-diverse communities of actinobacteria. Extremophiles.

[58] Polymenakou PN, Mandalakis M, Stephanou EG, Tselepides A (2008). Particle size distribution of airborne microorganisms and pathogens during an intense African dust event in the eastern Mediterranean. Environ. Health Perspect..

[59] Wang X (2017). Grain-size distribution of Pleistocene loess deposits in northern Iran and its palaeoclimatic implications. Quat. Int..

[60] Spring S, Kämpfer P, Schleifer KH (2001). *Limnobacter thiooxidans *gen. nov., sp. nov., a novel thiosulfate-oxidizing bacterium isolated from freshwater lake sediment. Int. J. Syst. Evol. Microbiol..

[61] Makhdoumi A (2018). Bacterial diversity in south coast of Caspian Sea: culture-dependent and culture-independent survey. Casp. J. Environ. Sci..

[62] Lindh MV (2015). Transplant experiments uncover Baltic Sea basin-specific responses in bacterioplankton community composition and metabolic activities. Front. Microbiol..

[63] Shifteh Som’e B, Ezani A, Tabari H (2012). Spatiotemporal trends and change point of precipitation in Iran. Atmos. Res..

[64] Mansouri Daneshvar MR, Ebrahimi M, Nejadsoleymani H (2019). An overview of climate change in Iran: facts and statistics. Environ. Syst. Res..

[65] Nercessian O, Noyes E, Kalyuzhnaya MG, Lidstrom ME, Chistoserdova L (2005). Bacterial populations active in metabolism of C_1_ in the sediment of Lake Washington, a freshwater lake. Appl. Environ. Microbiol..

[66] Caporaso JG (2012). Ultra-high-throughput microbial community analysis on the Illumina HiSeq and MiSeq platforms. ISME J.

[67] Takai K, Horikoshi K (2000). Rapid detection and quantification of members of the archaeal community by quantitative PCR using fluorogenic probes. Appl. Environ. Microbiol..

[68] Edgar RC, Flyvbjerg H (2015). Sequence analysis error filtering, pair assembly and error correction for next-generation sequencing reads. Bioinformatics.

[69] Maarastawi SA, Frindte K, Linnartz M, Knief C (2018). Crop rotation and straw application impact microbial communities in Italian and Philippine soils and the rhizosphere of *Zea mays*. Front. Microbiol..

[70] R Core Team. R: A language and environment for statistical computing version 3.2.5. Vienna: R Foundation for Statistical Computing. (2016).

[71] Oksanen, J. *et al.* Vegan: community ecology package. R package version 2.0-10 (2013).

