# Supplementary material for: Evidence for signatures of ancient microbial life in paleosols
Source: Sci Rep. 2020 Oct 8;10:16830. doi: 10.1038/s41598-020-73938-9 (PMC7545160; doi:10.1038/s41598-020-73938-9)
Supplement: Supplementary file 1 — Supplementary file1. [file 41598_2020_73938_MOESM1_ESM.pdf]

Supplementary material

Evidence for signatures of ancient microbial life in paleosols

Katharina Frindte[1], Eva Lehndorff[2], Stefan Vlaminck[3], Katharina Werner[1], Martin Kehl[3], Farhad Khormali[4], Claudia Knief[1]

[1] University of Bonn, Molecular Biology of the Rhizosphere, Nussallee 13, 53115 Bonn

[2] Bayreuth University, Soil Ecology, Dr.-Hans-Frisch-Str. 1-3, 95448 Bayreuth

[3] University of Cologne, Institute of Geography, Albertus Magnus Platz, 50923 Köln

[4] Gorgan University of Agricultural Sciences and Natural Resources, Department of Soil Sciences, Gogan, Iran

Corresponding author: kfrindte@uni-bonn.de

1. Supplementary tables

Table S1: Characteristics of the analyzed soil samples that were included in this study; data compiled from previous studies. Data include estimated soil age[1], soil horizon designation and interpretation of the degree and type of soil development (synsedimentary, postsedimentary) as well as inferred climatic conditions during the respective periods of increased dust accumulation (C horizons) and soil formation[2,3].

Color code contains information about the degree of soil development (loess, weak, moderate and strong development) and climatic conditions (arid, semiarid, subhumid and humid):
light green: horizons of the modern soil rich in organic matter;
brown: moderately developed horizons of synsedimentary paleosols formed under semiarid climate conditions;
yellow: weakly developed synsedimentary paleosol horizons formed under arid to semiarid conditions;
white: loess horizons with very limited pedogenic alteration;
blue: more humid climatic conditions during formation of postsedimentary paleosols.

Nr.	Depth [m]	Estimated age [kyr] and age classification	Soil horizon	Degree and type of soil development	Climatic condition
1	0.20	< 22.4 ± 1.6 (modern topsoil)	Ah	Strongly developed postsedimentary	Subhumid
2	0.35	< 22.4 ± 1.6 (modern subsoil)	ABwk	Strongly developed postsedimentary	Subhumid
3	1.27	ca. 22.4 ± 1.6 (modern subsoil)	CB/CBk	Weakly developed synsedimentary with postsedimentary overprint	Arid (to semiarid)
4	2.13	ca. 25.1 ± 1.7 (loess)	C	Loess with very limited pedogenetic alteration	Arid
5	2.89	26.0 ± 1.6 (paleosol)	CBk	Moderately developed synsedimentary	Arid (to semiarid)
6	3.95	26.0 ± 1.6 to 26.5 ± 1.8 (paleosol)	CBk	Weakly developed synsedimentary	Arid
7	5.10	29.7 ± 1.9 (paleosol)	C	Loess with very limited pedogenetic alteration	Arid
8	6.11	33.8 ± 2.2 (paleosol)	CBk	Weakly developed synsedimentary	Arid
9	7.31	33.8 ± 2.2 to 37.6 ± 2.4 (paleosol)	CBk	Weakly developed synsedimentary	Arid
10	8.28	37.6 ± 2.4 to 39.1 ± 2.4 (paleosol)	CBk	Weakly developed synsedimentary	Arid
11	9.72	39.1 ± 2.4 to 44.1 ± 2.7 (paleosol)	CBk	Moderately developed synsedimentary	Arid (to semiarid)
12	10.01	39.1 ± 2.4 to 44.1 ± 2.7 (paleosol)	CBk	Weakly developed synsedimentary	Arid
13	11.18	44.1 ± 2.7 to 52.4 ± 3.3 (paleosol)	CBk	Weakly to moderately developed synsedimentary	Arid
14	11.49	44.1 ± 2.7 to 52.4 ± 3.3 (loess)	C	Loess with very limited pedogenetic alteration	Arid
15	12.17	55.7 ± 4.2 (paleosol)	CBk	Loess with very limited pedogenetic alteration	Arid
16	12.65	57.1 ± 3.5 (loess)	C	Loess with very limited pedogenetic alteration	Arid
17	14.37	57.1 ± 3.5 to 57.9 ± 3.5 (loess)	C	Loess with very limited pedogenetic alteration	Arid

Nr.	Depth [m]	Estimated age [kyr] and age classification	Soil horizon	Degree and type of soil development	Climatic condition
18	15.77	66 ± 4.4 (paleosol)	CBk	Weakly developed synsedimentary	Arid
19	16.05	66 ± 4.4 to 61 ± 3.5 (paleosol)	BCk	Moderately developed synsedimentary	Semiarid
20	16.53	66 ± 4.4 to 61 ± 3.5 (paleosol)	CBk	Weakly developed synsedimentary	Arid
21	17.45	61 ± 3.5 to 74.9 ± 5 (paleosol)	BCk	Moderately developed synsedimentary	Semiarid
22	18.69	70.1 ± 4.3 (loess)	C	Loess with very limited pedogenetic alteration	Arid
23	19.55	74 ± 4.2 (paleosol)	Bwk	Moderately developed postsedimentary	Subhumid
24	19.95	74 ± 4.2 to 88.6 ± 5.7 (paleosol)	CBk	Weakly developed synsedimentary	Arid
25	21.53	97.3 ± 6.3 (paleosol)	Bwk	Moderately developed postsedimentary	Subhumid
26	21.90	98.1 ± 5.3 (paleosol)	Bt	Strongly developed postsedimentary	Subhumid (to humid)
27	22.65	98.1 ± 5.3 to116 ± 8 (paleosol)	Bkkm	Strongly developed postsedimentary	Subhumid (to humid)
28	23.01	116 ± 8 to 118 ± 8 (paleosol)	CBk	Weakly developed synsedimentary	Arid
29	23.93	104 ± 7 (paleosol)	Bwk	Moderately developed postsedimentary	Subhumid
30	24.99	104 ± 7 to 127 ± 8 (paleosol)	CBk	Weakly developed synsedimentary	Arid
31	25.55	< 127 ± 8 (paleosol)	Bwk	Moderately developed postsedimentary	Subhumid

Table S2: Results of Envfit analysis, used to correlate bacterial community compositional data with characteristics and soil properties of the different horizons in the profile (n=31). Values written in italics indicate *P*-values that were not significant after application of Bonferroni-Holm correction.

Parameter	R^2	*P*
**Depth**	0.80	< 0.001
**Age**[1]	0.81	< 0.001
**DNA content**	0.77	< 0.001
**Median grain size**[3§]	0.34	0.005
**Clay content**[3]	0.24	*0.027*
**Redness index**[3]	0.30	*0.012*
**Magnetic susceptibility**[2]	0.40	0.003
**Frequency dependent magnetic susceptibility**[2]	0.28	0.008
**pH**[§]	0.13	0.160
**Total organic carbon***	0.79/ 0.31	< 0.001/ 0.088
**Nitrogen***	0.78/ 0.33	< 0.001 / 0.077

[§] n=29 samples in this analysis (two samples were missing)

* For TOC and N, the first given value was calculated based on all samples, while the second value was calculated after the exclusion of modern soils to demonstrate that the correlations observed for these parameters resulted primarily due to differences between modern top soil versus buried horizons. These analyses are based on only 19 samples for TOC and N, because of missing values for some samples.

[1-3] References in which data of soil properties have been published.

Table S3: Pearson correlation coefficients of measured soil parameters with estimated values for richness, evenness and 16S rRNA gene copy numbers of the microbial community (n = 31). Significance levels are indicated by ** $P < 0.01$ and *** $P \leq 0.001$. Values written in italics indicate P-values that were not significant after application of Bonferroni-Holm correction.

	**Chao1**	**Evenness**	**Copy number**
**Depth**	-0.71**	-0.41*	-0.63**
**Age**	-0.61***	*0.37*	-0.73***
**DNA content**	0.94***	*0.44*	0.66***
**Median grain size**[§]	-0.52	-0.17	0.22
**Clay content**	0.27	0.09	-0.29
**Redness index**	0.46**	0.56**	0.03
**Magnetic susceptibility**	0.62***	0.53**	0.22
**Frequency dependent magnetic susceptibility**	0.48**	0.56**	0.15
**pH**[§]	-0.28	0.06	-0.26
**Total organic carbon**[#]	0.92***/0.67**	0.40/0.1	0.43/0.41
**Nitrogen content**[#]	0.89***/0.57*	0.369/0.02	0.41/0.49

[§] n=29 samples in this analysis (two samples were missing)

[#] For TOC and N, the first given value was calculated based on all samples, while the second value was calculated after the exclusion of modern soils to demonstrate that the correlations observed for these parameters resulted primarily due to differences between modern top soil versus buried horizons. These analyses are based on only 19 samples for TOC and N, because of missing values for some samples.

Table S4: Read numbers in the OTU table after bioinformatics processing and after removal of false positive reads (possibly derived from contaminations, as concluded from extraction and PCR blanks).

**Sample number**	**Reads after sequence quality control**	**High quality reads after false positive filtering**
1	27,186	26,670
2	14,633	14,293
3	24,959	24,638
4	20,662	20,457
5	42,254	41,580
6	39,569	39,144
7	26,254	25,583
8	39,984	39,217
9	26,720	26,477
10	33,893	33,539
11	17,765	17,638
12	22,203	20,515
13	27,011	26,842
14	35,116	33,779
15	12,919	12,812
16	26,281	26,134
17	20,344	19,977

18	28,503	28,076
19	30,419	30,244
20	11,098	10,946
21	19,051	18,453
22	18,165	18,023
23	13,806	13,565
24	19,652	19,388
25	13,089	12,754
26	4,718	4,121
27	11,426	10,500
28	12,220	11,630
29	26,837	26,466
30	14,070	13,899
31	6,839	6,534
extraction blank1	2,335	
extraction blank2	572	
extraction blank3	1,030	
PCR negative control	3,071	

2. Supplementary Figures

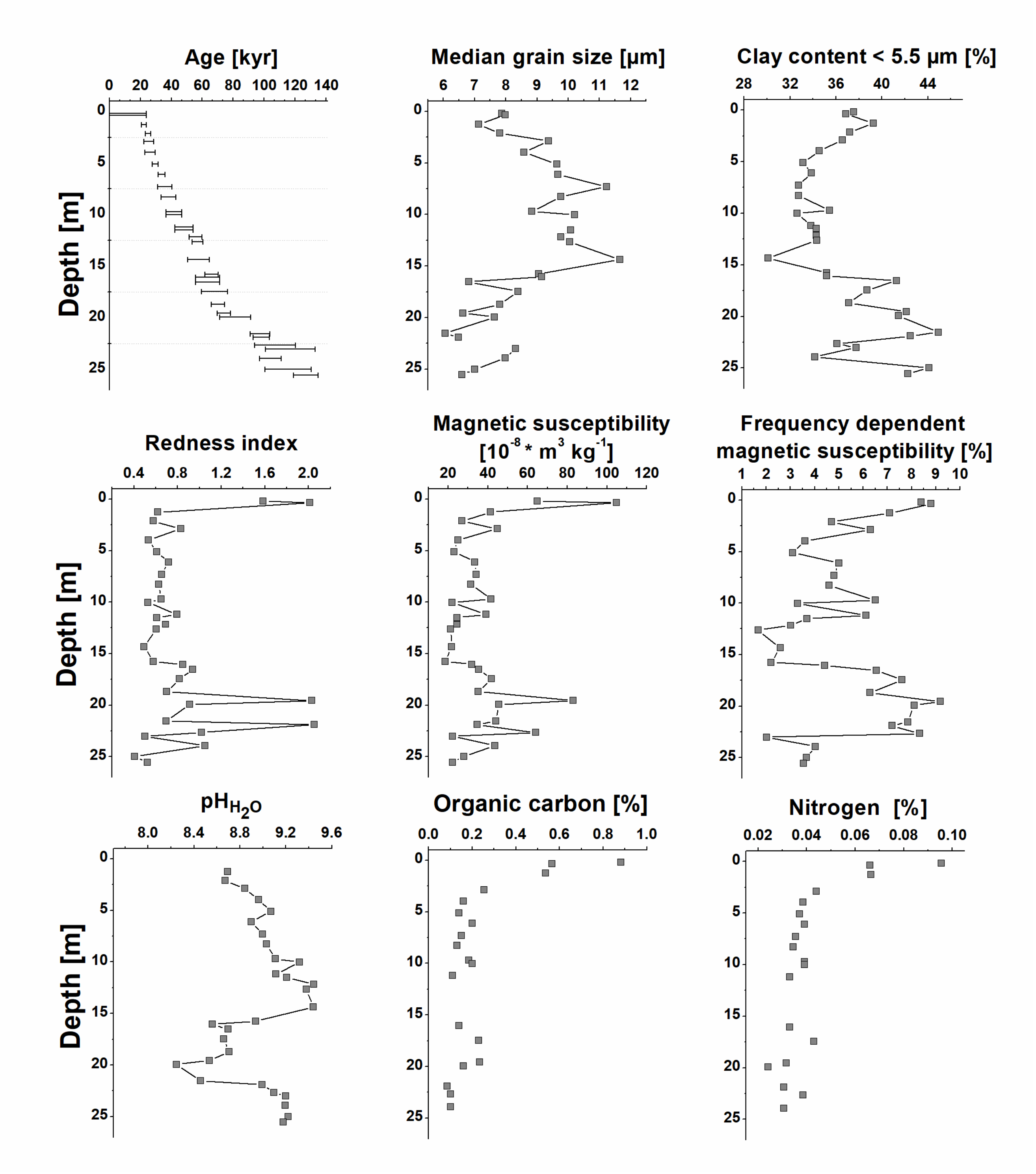

Figure S1: Changes of different environmental parameters with increasing depth. Age[1], volumetric median of grain size distribution[3], clay content[3]; redness index[3], mass specific magnetic susceptibility[2], frequency dependent magnetic susceptibility[2], pH in H_2O, total organic carbon and nitrogen.

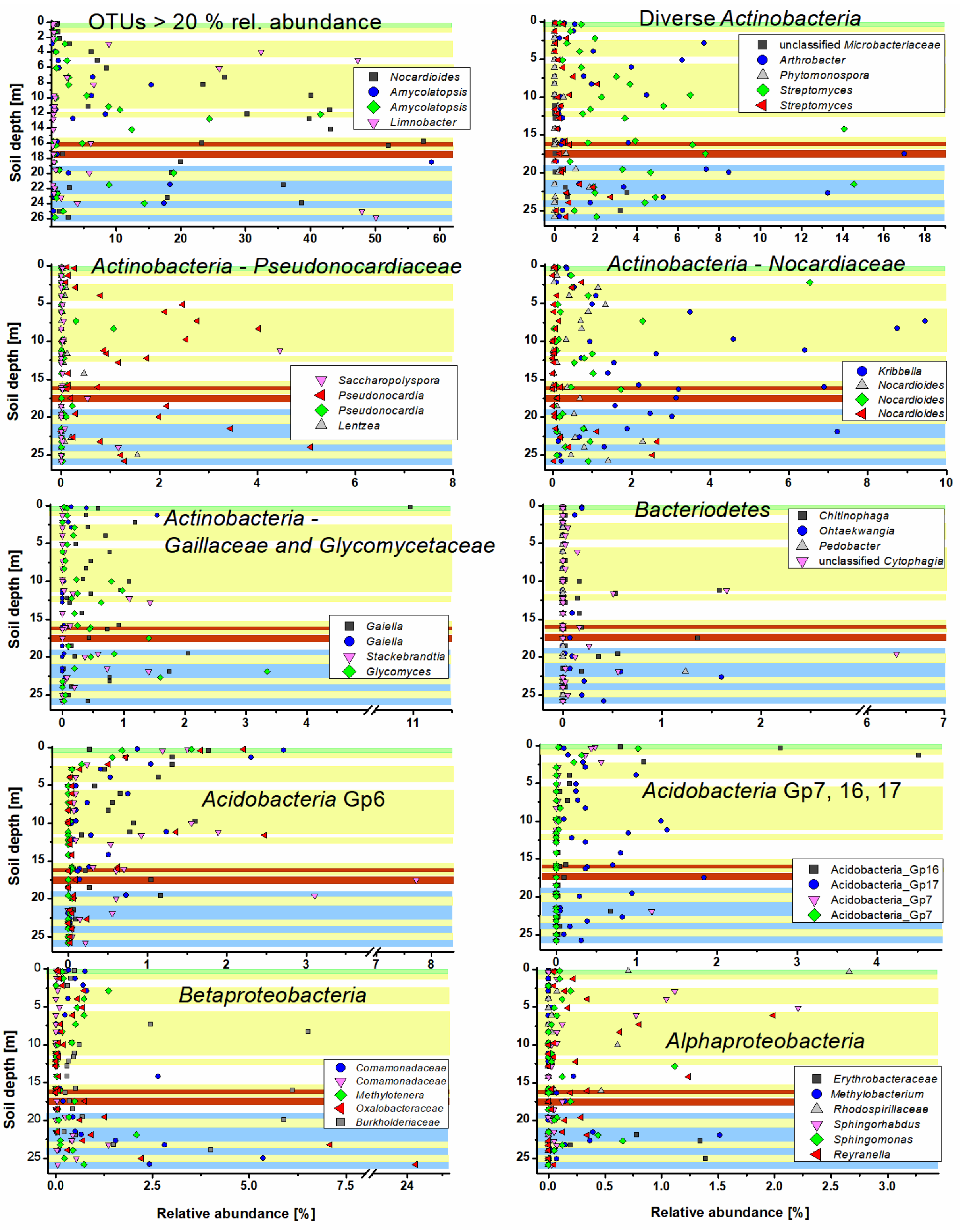

Figure S2: Selected high abundant OTUs (> 1% relative abundance) and their changes in relative abundance with increasing soil depth. The given phylogenetic classification corresponds to the last classifiable taxonomic rank of each OTU. Color code indicates soil horizons of the same type as defined in table S2.

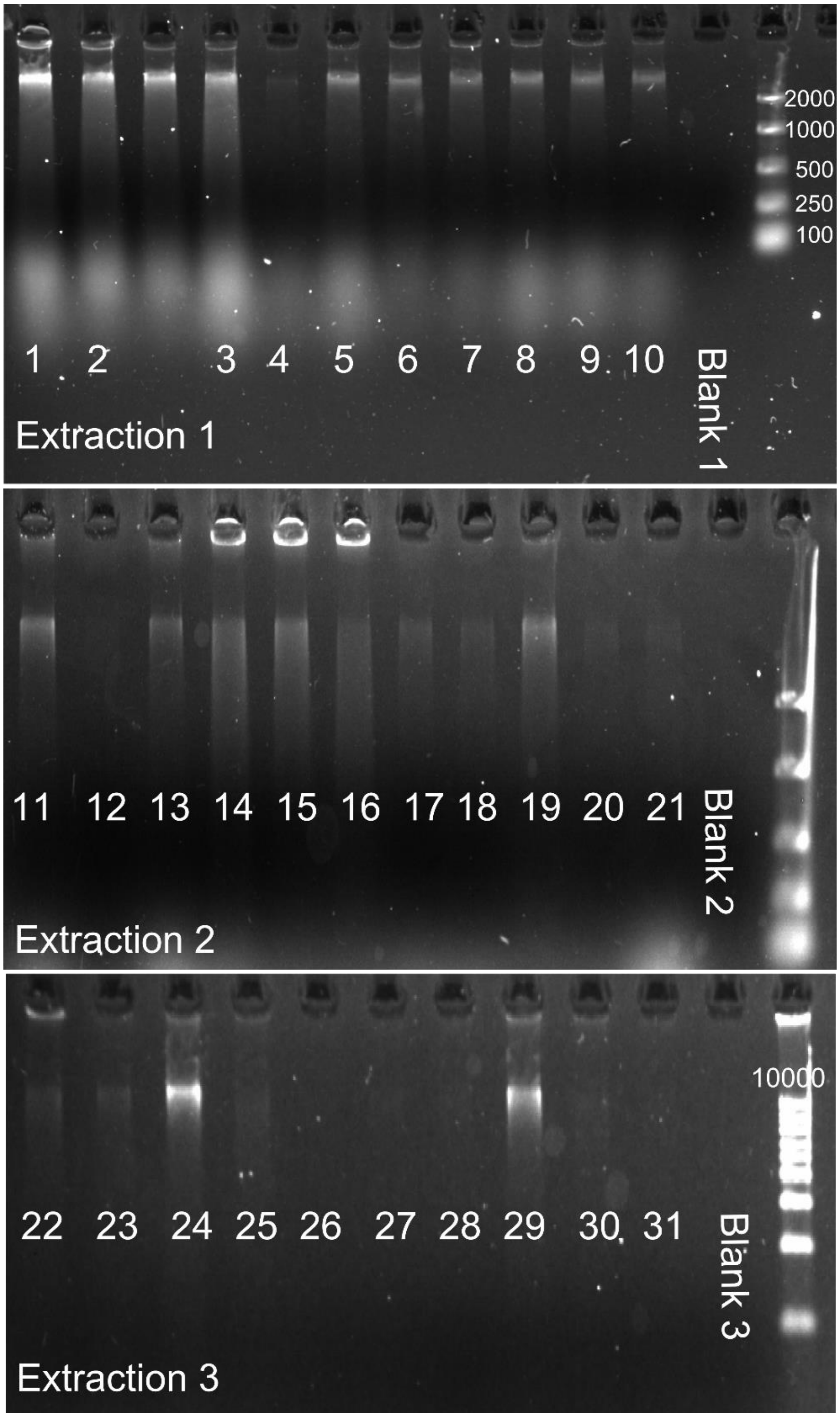

Figure S3: DNA extracts (before further purification) loaded onto 1.5% agarose gels. DNA extraction from soil samples was performed in three runs and included an extraction blank for each run. For the gels of extractions 1 + 2 a DNA ladder with a range from 100 – 2000 bp was used. The DNA ladder used for the gel picture of extraction 3 had a range from 100 – 10,000 bp.

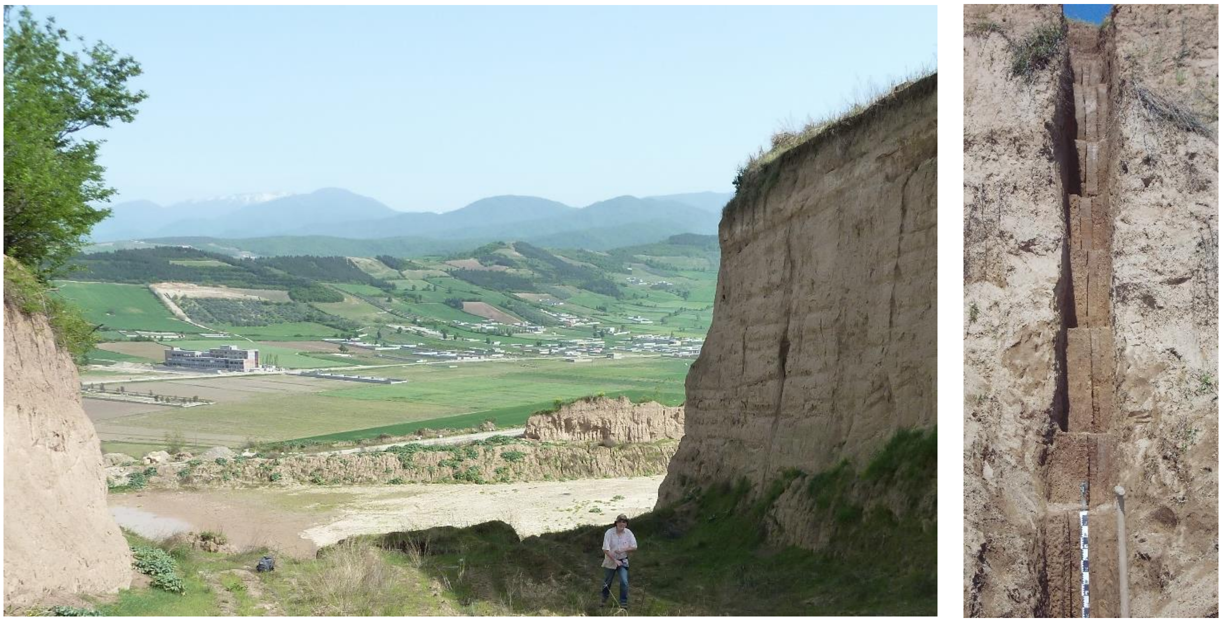

Figure S4: Loess covered foothills of Alborz Mountains at the sampling site at Toshan, Northern Iran (left site). Sampling was conducted along vertical sampling columns after digging steps into the loess-paleosol soil sequences (right site).

3. Supplementary information on methodology

Additional information about the soil sampling procedure

For soil sample collection, several staircases were prepared by digging about 0.5 to 3.5 m deep into vertical exposure walls of a loess paleosol sequence at Toshan, Northern Iran (Figure S4). Sampling was carried out at the cleaned surface of each step riser, from where one sample of ~100 g per 2 cm profile depth was extracted along a vertical column using a metal spatula. Samples from the center of each soil horizon were selected for this study, therewith keeping cross-contamination between layers minimal. Samples were stored in plastic bags until homogenization. Soils were dried completely at 40°C and homogenized by sieving through a 2 mm sieve and gentle manual milling with a mortar. They were stored dry and in the dark until further processing.

Considering the potential impact of the sample collection from different staircases, a specific separation of samples from the different staircases was not evident from the NMDS plot. Likewise, pronounced differences in the samples representing transitions from one staircase to the next one were not evident in Figure 4.

Investigation of former climatic conditions in the loess-paleosol soil sequence

The climatic information that was provided as description to each sample (Table S2) is the result of several studies investigating the former climate in this loess-paleosol profile and others found in this region[2–5]. The different paleosol horizons developed under different climatic conditions such as precipitation and vegetation growth, and thus showed different characteristics, which can be used to reconstruct past climate[6,7]. In brief, when only chemically unaltered loess was found, climate was dry, i.e. no chemically significant soil formation appeared. If a weathering of the mineral assemblage of the loess was apparent (abbreviated as B horizon, also in combination with C, and as reported by color, magnetic properties, change in grain size etc., see citations above), climate was moist enough to enable vegetation growth to at least steppe vegetation. One step further in soil formation, weathered minerals start to move downwards to form a clay enriched subsoil horizon (Bt horizon), which is only possible if precipitation exceeds evaporation rates, indicating a longer period of moist climate. Under such conditions forest is the most likely vegetation. Thus, the reconstruction of past climatic conditions is mainly based on the close correlation between paleosol features and vegetation cover, established in numerous investigations on climate drivers of soil formation on loess deposits of the study area and in other loess distribution areas. Weathering and soil formation can be quantitively described based on different physical or chemical soil properties, which can therewith serve as proxy for paleoclimatic conditions.

Water content of soils

After burial, the water content of the deeper layers has remained low, because in dry periods, rainfall and percolation of soil water into deeper layers is much reduced. Nowadays, the wetting front of the wet season travels down to about 1.0 m as indicated by formation of a carbonate enrichment horizon at this depth. Water content measured in sediment samples extracted for luminescence dating did not exceed 15 % [w/w] (measured for deeper soil horizons) and the

groundwater table is beyond 25 m below surface. Capillary rise from the groundwater is thus not possible. The loess-paleosol soil sequence does not show any feature that may point to a formerly higher groundwater table. The low water content and limited penetration of water enabled us to collect the samples via staircase profiles along the brim without a major risk for detecting present-day vertically translocated microorganisms in the MCs.

DNA extraction and purification

For DNA extraction, 3.75 g of soil was weighed into a 15-mL reaction tube and 5.25 g of zirconia beads were added, followed by 2.5 mL of CTAB buffer (10 % CTAB in 240 mM potassium phosphate buffer, pH 8.0) and 0.5 mL of sodium metaphosphate solution (0.1 M) as well as 0.3 mL of 10 % sodium dodecyl sulfate and 0.3 mL of 10 % N-laurosyl sarcosine. For phase extraction 3 mL phenol-chloroform isoamyl (25:24:1) were added. For cell lysis, the tubes were shaken in a FastPrep-96 at 1200 rpm for 1 min (MP Biomedicals, Eschwege, Germany). Thereafter, tubes were centrifuged at 20,124 x g at 4 °C for 10 min. The upper phase was transferred to a new 15 ml tube and 3 mL phenol-chloroform-isoamyl was added again. Phases were mixed by inversion and centrifuged as before. The upper phase was transferred to a new 15 ml tube and 2 volumes of polyethylene glycol solution (20% PEG 8000 in 2.5 M NaCl) were added for DNA precipitation. Tubes were incubated for 2 h at room temperature and finally centrifuged for 60 min at 4 °C at 20,124 x g. The resulting pellet was washed with 5 ml iced 70 % ethanol. Ethanol was removed after centrifugation (10 min, 4 °C, max. speed), the pellets were air dried and DNA dissolved in 300 µL of PCR-grade water. The extraction was performed in three runs, resulting in three extractions blanks, which were included in further molecular analyses.

Prior to PCR, 150 µl of the DNA extract underwent further purification. A first purification was done with magnetic beads (HighPrep beads, Biozym) according to the manufacturer’s instructions, except that 35 µL of bead solution and 70 µL of PEG buffer (20% PEG 8000 in 2.5 M NaCl) were added. DNA was eluted in 25 µL of PCR-grade water. This DNA suspension, which lost DNA fragments <400 bp after purification, was used for a second purification using the NucleoSpin gDNA Clean-Up kit (Macherey-Nagel, Düren, Germany) with final elution of the DNA in 50 µL of PCR-grade water. DNA was stored at -20°C.

Amplicon PCR and Library preparation

PCRs for Illumina amplicon sequencing were performed with primer pair 515F/806R[8] A first PCR was performed with 30 cycles in triplicates per sample. The triplicates were pooled for the second PCR with barcoded primers (5 cycles). The reaction mixture contained 0.2 – 2.0 ng µL^{-1} DNA template in the first round and 2 µL of PCR template in the second round. The 10 µl PCR assays contained 0.2 µM of each primer, 200 µM of each dNTP, polymerase-supplied buffer II, 1 mM $MgCl_2$, 0.8 µg µL^{-1} bovine serum albumine and 0.4 µL (first round) or 1 µL (second round) of AccuPrime DNA polymerase (Thermo-Fisher Scientific, Darmstadt, Germany). Further details about PCR program and primer barcoding strategy have been described earlier[9].

Library preparation with the purified and pooled PCR products have been performed at the Max Planck Genome Center Cologne, Germany (https://mpgc.mpipz.mpg.de/home/). 100 ng genomic DNA has initially been used for library preparation with NEBNext Ultra II DNA Library Prep Kit

for Illumina (New England Biolabs). Library preparation included 4 cycles of PCR amplification. Quality and quantity were assessed at all steps via capillary electrophoresis (TapeStation, Agilent Technologies) and fluorometry (Qubit, Thermo Fisher Scientific).

qPCR

The abundance of bacteria was determined by quantitative PCR (qPCR) using the primer system (Bac349f/Bac806r)[10]. Ten-µL PCR assays with 1 µL of 10-fold diluted DNA template were prepared using the SsoAdvanced™ Universal SYBR Green Supermix according to the manufacturer's instructions (Bio-Rad, Munich, Germany). The quantification of each sample was performed in technical duplicates using a CFX Connect™ Real-Time PCR Detection System (Bio-Rad, Munich, Germany). The thermal cycling protocol consisted of an initial denaturation step at 95 °C for 2 min, followed by 40 cycles at 95 °C for 10 s and 62 °C for 45 s, including the fluorescence quantification step. Data were normalized to gene copy numbers per g dry soil. PCR products were checked for the correct length via melt curve analysis and gel electrophoresis. As standard we used an almost complete 16S rRNA gene PCR product amplified with primers 9f/1492r, which was cloned into a plasmid with the pGEM-T Easy Vector System (Promega, Mannheim, Germany). Standard dilutions ranged from 10^9 to 10^2 copies per reaction. Efficiency of the qPCR assays ranged between 0.89 and 0.95. Different dilutions of the DNA extracts were initially tested to exclude inhibition of the PCR assay, resulting in the use of a 10-fold diluted extract in the qPCR assay.

Correction for false positive results due to contamination

To correct for false positive OTUs derived from contamination of chemicals used for DNA extraction, blanks were included in each DNA extraction procedure. Moreover, a PCR blank was included during PCR. The weak positive PCR products from all these blanks underwent sequencing. After bioinformatics processing, the resulting OTU table was corrected for false positives. In a first step a NMDS plot was drawn to control if blanks differed markedly from the soil samples. The analysis showed that the soil samples were clearly distinct from all contamination blanks. In a further step, false-positive OTUs that were present in the PCR negative controls and the extraction blanks were identified (Table S5). For this purpose, a rarefaction was applied to adjust the read number of the soil samples to those of the controls, which allowed a better comparison. OTUs with similar or higher read numbers in blanks than in soil samples were excluded from the original non-rarefied dataset. 3% of the total reads or 1.8% of the OTUs were removed based on this correction (Tables S4 and S5). The OTU table corrected in this way was used for all further analyses including all soil samples and excluding controls after data rarefaction.

4. Supplementary results

Additional information about the distribution of taxa in the loess-paleosol sequence

The relative abundance of the majority of taxa was highly variable between the soil horizons. Members of the same genus or family did not necessarily show the same distribution patterns and abundant OTUs, e. g. some of the phylum *Actinobacteria* (e.g. *Amylocolatopsis*), were not highly abundant in all soil horizons (Figure S2). Characteristic for the upper (modern) soil horizons were for example members of the phylum *Acidobacteria* (members of GP6, GP7, GP16), *Actinobacteria* (*Gaiella*), *Verrucomicrobia* (*Spartobacteria* genera incertae sedis) and the class of *Alphaproteobacteria* (*Rhodospirillaceae*) (Figure S2). Moreover, no OTUs were consistently present in all paleosols, but some OTUs were specifically found in the paleosols that developed under more humid conditions (19.5, 21.5 - 22.65, 23.9 and 25.5 m), e.g. *Bacteriodetes* (*Ohtaekwangia*, *Pedobacter*), *Betaproteobacteria* (*Methylotenera* and members of the families *Oxalobacteraceae* and *Comamonadaceae*), *Alphaproteobacteria* (*Erythrobacteraceae*, *Methylobacterium*), *Actinobacteria* (*Phytomonospora* and OTUs representing *Nocardioides* and *Microbacteriaceae*).

5. References:

1. Lauer, T. *et al.* Luminescence-chronology of the loess palaeosol sequence Toshan, Northern Iran - A highly resolved climate archive for the last glacial-interglacial cycle. *Quat. Int.* **429**, 3–12 (2017).
2. Vlaminck, S. *et al.* Late Pleistocene dust dynamics and pedogenesis in Southern Eurasia - Detailed insights from the loess profile Toshan (NE Iran). *Quat. Sci. Rev.* **180**, 75–95 (2018).
3. Vlaminck, S. *et al.* Loess-soil sequence at Toshan (Northern Iran): Insights into late Pleistocene climate change. *Quat. Int.* **399**, 122–135 (2016).
4. Shahriari, A. *et al.* Biomarkers in modern and buried soils of semi-desert and forest ecosystems of northern Iran. *Quat. Int.* **429**, 62–73 (2017).
5. Lauer, T. *et al.* The Agh Band loess-palaeosol sequence - A terrestrial archive for climatic shifts during the last and penultimate glacial - interglacial cycles in a semiarid region in northern Iran. *Quat. Int.* **439**, 13–30 (2017).
6. Khormali, F. & Kehl, M. Micromorphology and development of loess-derived surface and buried soils along a precipitation gradient in Northern Iran. *Quat. Int.* **234**, 109–123 (2011).
7. Khormali, F., Ghergherechi, S., Kehl, M. & Ayoubi, S. Soil formation in loess-derived soils along a subhumid to humid climate gradient, Northeastern Iran. *Geoderma* **179**–**180**, 113–122 (2012).
8. Caporaso, J. G. *et al.* Ultra-high-throughput microbial community analysis on the Illumina HiSeq and MiSeq platforms. *ISME J* **6**, 1621–1624 (2012).
9. Frindte, K., Pape, R., Werner, K., Löffler, J. & Knief, C. Temperature and soil moisture control microbial community composition in an arctic–alpine ecosystem along elevational and micro-topographic gradients. *ISME J.* **13**, 2031–2043 (2019).
10. Takai, K. & Horikoshi, K. Rapid detection and quantification of members of the archaeal community by quantitative PCR using fluorogenic probes. **66**, 5066–5072 (2000).
